# Supplementary material for: Have sedentary lifestyles reached even remote parts of the Global South? Evidence from school-going adolescents’ time use in India
Source: PLoS One. 2026 Feb 20;21(2):e0338096. doi: 10.1371/journal.pone.0338096 (PMC12922976; doi:10.1371/journal.pone.0338096)
Supplement: S1 Fig — (DOCX) [file pone.0338096.s002.docx]

**Supplementary Figure 1:** 24-hour Time-Use Data Collection Instrument

We would like to know about what you do during a day. Let us talk about yesterday. Please tell me everything you did from the time you woke up in the morning to the time you went to sleep at night. I would also like to know how long each activity took, where you were when you did it, who you were with, and what else you were doing at the same time.

Here is an example of a day given by someone your age.

| TIME | A | B | C | D | E | F | G |
| --- | --- | --- | --- | --- | --- | --- | --- |
| *Midnight* | *What did you do?* | *Time Began* | *Time Ended* | *Where were you?* | *Who was doing this with you?* | *Who else was in the same room but not doing the same thing?* | *What else were you doing at the same time?* |
|  | 1. Sleeping | 12:00 | 7:10 | At home | ---------------- | ------------------ | ------------------ |
|  | Trying to wake up | 7:10 | 7:20 | At home | ----------------- | ------------------ | ------------------- |
|  | Showering | 7:20 | 7:35 | At home | ----------------- | ------------------ | -------------------- |
|  | Getting dressed | 7:35 | 7:40 | At home | ----------------- | ------------------- | ------------------ |
|  | 2. Eating breakfast | 7:40 | 7:55 | At home | Mother, sister | X | Talking, watching TV |
|  | 3. Driving to school | 7:55 | 8:05 | In car | X | X | Listening to CDs |
|  | 4. At school | 8:05 | 2:35 | At school | -------------- | ------------------ | ----------------- |
|  | Going to track practice | 2:35 | 2:45 | Walking | Friends, teammates | Other kids | Talking, goofing around |
|  | Changing into track uniform | 2:45 | 3:00 | Field locker room | ---------------- | ------------------ | ------------------- |
|  | Track practice | 3:00 | 4:15 | High school’s track field | Friends, teammates, coaches | Other friends, teammates, coaches | X |
|  | Going home | 4:15 | 4:25 | In car | X | X | Listening to CD’s |
|  | 5. Eating dinner | 4:25 | 5:00 | At home | X | X | Checking email, using IM’s |
| 5 P.M. | Playing computer | 5:00 | 5:20 | At home | X | Sister | Chatting, playing games |

Now think about yesterday. DAY OF THE WEEK:_______________

Please complete the table, indicating all activities you did yesterday, for the entire day. Don’t leave anything out!

| A | B | C | D | E | F | G |
| --- | --- | --- | --- | --- | --- | --- |
| *What did you do?* | *Time Began* | *Time ended* | *Where were you?* | *Who was doing this with you?* | *Who else was in the same room but not doing the same thing?* | *What else were you doing at the same time?* |
|  | Midnight |  |  |  |  |  |
|  |  |  |  |  |  |  |
|  |  |  |  |  |  |  |
|  |  |  |  |  |  |  |
|  |  |  |  |  |  |  |
|  |  |  |  |  |  |  |
|  |  |  |  |  |  |  |
|  |  |  |  |  |  |  |
|  |  |  |  |  |  |  |
|  |  |  |  |  |  |  |
|  |  |  |  |  |  |  |
|  |  |  |  |  |  |  |
|  |  |  |  |  |  |  |
