# Supplementary material for: Have sedentary lifestyles reached even remote parts of the Global South? Evidence from school-going adolescents’ time use in India
Source: PLoS One. 2026 Feb 20;21(2):e0338096. doi: 10.1371/journal.pone.0338096 (PMC12922976; doi:10.1371/journal.pone.0338096)
Supplement: S2 Table — (DOCX) [file pone.0338096.s003.docx]

**Supplementary Table 2:** Codebook for Categorizing Sedentary Activities

| **Code #** | **Activity** | **Description** |
| --- | --- | --- |
|  | **Passive** |  |
| 0 | Going to bed or waking up | Lying in bed not yet asleep or trying to get up |
| 3 | Watching someone work or do tasks | Passive watching without helping |
| 27 | Doing nothing | Doing nothing, thinking, waiting |
|  | **School and learning** |  |
| 60 | Sitting in class at school | Sitting in the classroom with minimal movement |
| 61 | Tutoring | Receiving tuition |
| 62 | Homework | Studying at home or elsewhere outside of class time, doing research, reviewing homework with parent |
| 63 | Training | Being taught other than school, e.g. apprenticeship |
| 64 | Meetings | Attended meeting or assembly in school, youth group, other |
|  | **Play and social activities** |  |
| 70 | Playing on computer or mobile | Includes games, chatting, texting, Facebook |
| 71 | Reading and writing | For pleasure or for school work |
| 75 | Non-active game | Chess, Carrom, card games, guessing games |
| 77 | Watching games | Watching others play or an organized game, or parade, fair, not on TV |
| 78 | Watching TV | Watching TV or movies |
|  | **Travel** |  |
| 81 | Sitting or standing while traveling | Waiting for bus, sitting in car or on bus |
