# Supplementary material for: Have sedentary lifestyles reached even remote parts of the Global South? Evidence from school-going adolescents’ time use in India
Source: PLoS One. 2026 Feb 20;21(2):e0338096. doi: 10.1371/journal.pone.0338096 (PMC12922976; doi:10.1371/journal.pone.0338096)
Supplement: S3 Table — (DOCX) [file pone.0338096.s004.docx]

**Supplementary Table 3.** Duration of sedentary activity bouts among school-going adolescents in Vijayapura, India by gender and SES, among those engaging in each activity

|  | **Mean minutes/day (SD) among participants** | | | | | | |
| --- | --- | --- | --- | --- | --- | --- | --- |
| **Activity** | **Total** | **Boys (n=197)** | **Girls (n=198)** | **p** | **Public school** | **Private school** | **p** |
|  | **Mean (SD)** | **Mean (SD)** | **Mean (SD)** |  | **Mean (SD)** | **Mean (SD)** |  |
| **School/Learning Domain** | 417.75 (160.61) | 428.05 (152.75) | 407.41 (167.90) |  | 350.94 (149.30) | 484.22 (143.22) | *** |
| Sitting in class at school | 274.36 (90.40) | 277.54 (96.72) | 271.12 (83.65) |  | 233.56 (80.57) | 303.67 (85.77) | *** |
| Being tutored at school | 121.10 (67.68) | 107.47 (51.01) | 141.17 (83.40) | * | 98.15 (44.81) | 131.10 (73.60) | * |
| Doing homework | 163.12 (92.45 | 161.87 (93.86) | 165.49 (91.24) |  | 151.90 (92.06) | 175.11 (91.67) | * |
| Being taught outside of school | 43.34 (61.46) | 48.69 (70.34) | 38.22 (50.04) |  | 55.01 (70.45) | 32.59 (49.67) | ** |
| Attending meetings | 46.28 (42.27) | 51.20 (44.67) | 42.05 (40.04) |  | 50.89 (44.75) | 35.13 (33.64) |  |
| **Leisure/Social Domain** | 137.50 (112.54) | 133.17 (109.58) | 141.62 (115.46) |  | 135.62 (109.41) | 139.39 (115.89) |  |
| Playing on computer or mobile | 56.94 (47.76) | 60.75 (52.95) | 55.0 (36.97) |  | 56.0 (45.13) | 57.81 (51.57) |  |
| Reading and writing | 161.74 (130.26) | 166.26 (130.65) | 157.21 (131.30) |  | 171.75 (115.74) | 154.23 (140.91) |  |
| Watching games | 147.50 (79.10) | 195.00 (21.21) | 123.75 (89.57) |  | 147.50 (79.10) | 0.00 (0.00) | - |
| Watching TV | 97.84 (69.67) | 88.89 (59.50) | 106.21 (77.25) | * | 98.24 (69.40) | 97.43 (70.20) |  |
| **Travel Domain** | 83.20 (76.46) | 93.36 (89.63) | 74.20 (61.67) |  | 106.07 (110.32) | 81.10 (72.72) |  |
| Sitting or standing while traveling | 83.20 (76.46) | 93.36 (89.63) | 74.20 (61.67) |  | 106.07 (110.32) | 81.10 (72.72) |  |
| **Passive Domain** | 39.35 (42.75) | 35.55 (37.37) | 42.00 (46.21) |  | 47.78 (51.38) | 34.54 (36.46) |  |
| Lying in bed | 33.22 (33.78) | 29.73 (31.83) | 35.55 (35.06) |  | 32.88 (26.98) | 33.36 (36.45) |  |
| Watching someone work or do tasks | 71.79 (68.52) | 100.00 (65.57) | 64.09 (70.28) |  | 79.17 (71.44) | 27.50 (17.68) |  |
| Doing nothing, thinking, waiting | 36.67 (18.89) | 41.00 (17.46) | 15.00 (n/a) | - | 38.33 (18.93) | 35.00 (22.91) |  |

N.B. Mean duration calculated only from those who participated in each activity. Minutes/day compared across strata using independent samples t-test.

*p<0.05, **p<0.01, ***p<0.001.
